# Compensatory Plasticity in the Lateral Extrastriate Visual Cortex Preserves Audiovisual Temporal Processing following Adult-Onset Hearing Loss

**DOI:** 10.1155/2019/7946987

**Published:** 2019-05-15

**Authors:** Ashley L. Schormans, Brian L. Allman

**Affiliations:** Department of Anatomy and Cell Biology, Schulich School of Medicine and Dentistry, University of Western Ontario, London, Ontario, Canada

## Abstract

Partial hearing loss can cause neurons in the auditory and audiovisual cortices to increase their responsiveness to visual stimuli; however, behavioral studies in hearing-impaired humans and rats have found that the perceptual ability to accurately judge the relative timing of auditory and visual stimuli is largely unaffected. To investigate the neurophysiological basis of how audiovisual temporal acuity may be preserved in the presence of hearing loss-induced crossmodal plasticity, we exposed adult rats to loud noise and two weeks later performed *in vivo* electrophysiological recordings in two neighboring regions within the lateral extrastriate visual (V2L) cortex—a multisensory zone known to be responsive to audiovisual stimuli (V2L-Mz) and a predominantly auditory zone (V2L-Az). To examine the cortical layer-specific effects at the level of postsynaptic potentials, a current source density (CSD) analysis was applied to the local field potential (LFP) data recorded in response to auditory and visual stimuli presented at various stimulus onset asynchronies (SOAs). As predicted, differential effects were observed in the neighboring cortical regions' postnoise exposure. Most notably, an analysis of the strength of multisensory response interactions revealed that V2L-Mz lost its sensitivity to the relative timing of the auditory and visual stimuli, due to an increased responsiveness to visual stimulation that produced a prominent audiovisual response irrespective of the SOA. In contrast, not only did the V2L-Az in noise-exposed rats become more responsive to visual stimuli but neurons in this region also inherited the capacity to process audiovisual stimuli with the temporal precision and specificity that was previously restricted to the V2L-Mz. Thus, the present study provides the first demonstration that audiovisual temporal processing can be preserved following moderate hearing loss via compensatory plasticity in the higher-order sensory cortices that is ultimately characterized by a functional transition in the cortical region capable of temporal sensitivity.

## 1. Introduction

Following sensory deprivation, such as vision or hearing loss, the brain has the capacity to undergo extensive reorganization, which is often characterized by an increased responsiveness of neurons in the deprived sensory cortex to the spared senses (i.e., crossmodal plasticity) (for review, see [1]). For example, in conditions of profound hearing loss, the “deafened” auditory cortex has shown increased activity to visual and/or tactile stimuli, as measured using neuroimaging in humans as well as invasive electrophysiological recordings in animal models [2–6]. In addition to these neurophysiological changes, behavioral studies have also identified that deafness in early life can lead to improved performance on tasks that emphasize the processing of peripheral visual stimuli or visual motion [7–11].

Despite the high prevalence of *partial* hearing impairments in society (~1 out of 5 adults) [12, 13], appreciably less is known about the nature and extent of crossmodal plasticity that occurs in individuals who retain some level of residual auditory processing, compared to cases of profound hearing loss. That said, recent studies have confirmed that crossmodal plasticity does occur following mild-moderate hearing loss, albeit to a lesser degree than in deaf subjects. Interestingly, not only does the auditory cortex show increased visual and tactile responses following adult-onset hearing impairment in humans [14–16] and ferrets [17] but our recent work in rats exposed to loud noise found a differential effect in how auditory and visual stimuli were processed in the auditory cortex versus the multisensory cortex [18, 19]. More specifically, despite accounting for each rat's elevated hearing threshold two weeks postnoise exposure, we observed a decrease in the proportion of neurons in the multisensory cortex that could be activated by auditory stimuli, as well as an increased responsiveness to visual stimuli in both the auditory and multisensory cortices [18, 19]. Consequently, following noise exposure, the cortical area now showing the greatest relative degree of multisensory convergence transitioned beyond the audiovisual cortex into a neighboring auditory region—findings which led to the suggestion that crossmodal plasticity induced by adult-onset hearing impairment can manifest in higher-order areas as a transition in the functional border of the audiovisual cortex.

In normal-hearing subjects, there is clear evidence of several behavioral advantages afforded by the brain's natural ability to integrate auditory and visual information, including improved detection, localization, and identification of the stimuli. In addition, psychophysical testing has revealed that auditory and visual stimuli presented within ~100 ms offset from each other can be bound into a unified percept, with subjects showing difficulty in accurately judging whether the auditory or visual stimulus was presented first. Ultimately, because neuroimaging studies in humans have shown that synchronized activity in the multisensory cortex underlies audiovisual temporal acuity [20], it is reasonable to question how partial hearing impairment, and its ensuing crossmodal plasticity, could disrupt one's perception of the relative timing of audiovisual stimuli and ultimately the binding of these multisensory cues into a unified percept. Of the few reports available, however, it appears that audiovisual synchrony perception is largely preserved in hearing-impaired subjects [21–23], provided that potential confounding factors, such as aging, are addressed. Moreover, we recently reported that adult rats with a moderate hearing loss experienced a rapid recalibration of their ability to accurately judge the order of audiovisual stimuli, with temporal perception being restored two weeks following the loud noise exposure [24]. This inconsistency between the extent of crossmodal plasticity reported previously and the apparent lack of behavioral consequences raises an important question: how is the brain able to maintain (or re-establish) temporally precise audiovisual integration and perception in the presence of extensive sensory reorganization in the cortical regions thought to subserve such behavioral tasks?

To date, no studies have investigated whether changes in the temporal precision of audiovisual processing occur at the neuronal level following adult-onset hearing loss or if these crossmodal effects differ across the neighboring regions of the multisensory cortex that normally integrate audiovisual stimuli. Thus, in the present study, we used *in vivo* extracellular electrophysiological recordings in anesthetized rats to investigate how crossmodal plasticity induced by moderate hearing loss alters audiovisual temporal processing across the distinct layers of higher-order sensory cortices. To do so, adult rats were exposed to loud noise exposure, and two weeks later, extracellular electrophysiological recordings were performed within two neighboring regions of the lateral extrastriate visual (V2L) cortex—a multisensory area known to be responsive to audiovisual stimuli (V2L multisensory zone) and a more predominantly auditory area (V2L auditory zone). More specifically, a 32-channel linear electrode array was inserted perpendicular to the cortical surface and laminar processing was examined within each cortical region in response to combined audiovisual stimuli at various stimulus onset asynchronies (SOAs). To examine the layer-specific effects of crossmodal plasticity at the level of postsynaptic potentials, a current source density (CSD) analysis was applied to the local field potential (LFP) data. Based on our earlier work which suggested that moderate hearing loss caused an expansion of the functional boundary of the audiovisual cortex into the neighboring auditory regions, we predicted that the auditory zone of the V2L cortex would not only become more responsive to visual stimuli postnoise exposure but also inherit the capacity to process audiovisual stimuli with the temporal precision and specificity that was previously restricted to the audiovisual cortex in normal-hearing rats—electrophysiological results that could provide the neurophysiological basis for the preservation/restoration of audiovisual temporal perception following adult-onset hearing loss.

## 2. Methods

The present study included two experimental series—each using a separate group of adult male Sprague-Dawley rats (*n* = 34 total; Charles River Laboratories Inc., Wilmington, MA). Prior to examining the cortical consequences of noise-induced crossmodal plasticity, we conducted Experiment 1 to first confirm that the V2L cortex does indeed play an important role in audiovisual temporal acuity, by pharmacologically silencing the region in rats (*n* = 16) trained to perform perceptual judgment tasks. In Experiment 2, we then performed electrophysiological recordings in anesthetized rats (*n* = 18) to examine the effect of noise-induced crossmodal plasticity on audiovisual temporal processing within two regions of the V2L cortex. All experiments were approved by the University of Western Ontario Animal Care and Use Committee and were conducted in accordance with the guideline established by the Canadian Council of Animal Care.

### 2.1. Experiment 1: Role of V2L in Audiovisual Temporal Acuity

#### 2.1.1. Audiovisual Behavioral Tasks: TOJ and SJ

Using appetitive operant conditioning, rats were trained on a two-alternative forced-choice paradigm that assessed their ability to perform audiovisual temporal-order judgments (TOJ; *n* = 8) or synchrony judgments (SJ; *n* = 8). In the TOJ task, rats were trained to differentiate the temporal order of auditory and visual stimuli, whereas rats trained on the SJ task learned to differentiate between trials when the auditory and visual stimuli were presented synchronously or when the visual stimulus preceded the auditory stimulus (i.e., asynchronous). For both tasks, behavioral training began at 70 days old (body mass: 284 ± 7.0 g) and the rats were trained 6 days a week. All experimental testing took place when the rats were between 8 and 9 months of age.

Behavioral training and testing were conducted in a standard modular test chamber (ENV-008CT; Med Associates Inc., St. Albans, VT) that was housed within a sound-attenuating box (29” W by 23.5” H by 23.5” D; Med Associates Inc.). The front wall of the behavioral chamber included a nose poke as well as a left and right feeder trough, each fitted with an infrared detector to monitor the rat's performance. The test chamber was illuminated by a house light on the back wall. Real-time processing hardware (RZ6 and BH-32, Tucker Davis Technologies, Alachua, FL) were interfaced with the test chamber. Custom behavioral protocols running in MATLAB (EPsych Toolbox, http://dstolz.github.io/epsych/) monitored nose poke responses and controlled the presentation of the auditory and visual stimuli, as well as the positive reinforcement (i.e., sucrose pellet delivery) and punishment (i.e., turning off the house light and an inability to commence the next trial).

The auditory stimulus was a 50 ms noise burst (75 dB SPL; 1-32 kHz) presented from a speaker (FT28D, Fostex, Tokyo) mounted on the ceiling of the behavioral chamber near the front wall. The intensity of the auditory stimulus was calibrated using custom MATLAB software with a 1/4-inch microphone (2530, Larson Davis, Depew, NY) and preamplifier (2221; Larson Davis). The visual stimulus was a 50 ms light flash (27 lux) from an LED (ENV-229M; Med Associates Inc.) located above the center nose poke. An LED light meter (model LT45, Extech Instruments, Nashua, NH) was used to determine the intensity of the visual stimulus.

#### 2.1.2. Behavioral Training for the TOJ and SJ Tasks

**Figure 1:**
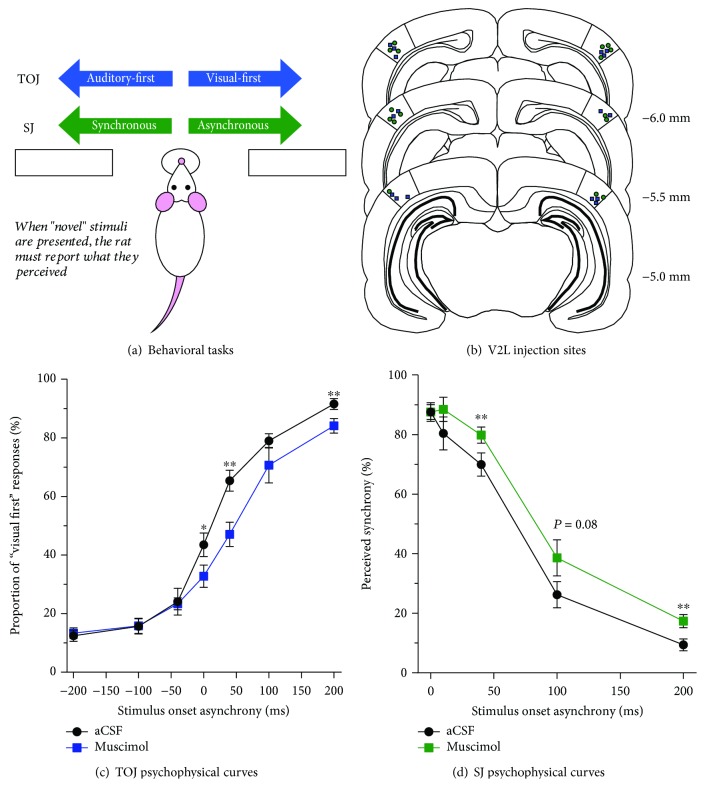
Pharmacological silencing of the V2L cortex disrupts audiovisual temporal acuity in rats. (a) An overview of both the TOJ task and the SJ task that were used to screen rats for their audiovisual temporal acuity. Across several stages, rats were trained to select the right or left feeder trough depending on the stimulus condition presented (i.e., TOJ task: auditory-first = left trough and visual-first = right trough; SJ task: synchronous = left trough and asynchronous = right trough). (b) Schematic of the location of the drug infusion cannulae reconstructed from histological sections for each of the rats trained on the TOJ (blue squares) or the SJ task (green circles). (c) Behavioral performance on the TOJ task was plotted as the proportion of trials perceived as visual first for test sessions completed following the infusion of aCSF (black circles) and muscimol (blue squares). Overall, there was a rightward shift in the TOJ psychometric curve when muscimol was infused into the V2L cortex, with a significant decrease in trials perceived as visual first at SOAs of 40 and 200 ms (^∗∗^*p* < 0.007), as well as a modest decrease at an SOA of 0 ms (^∗^*p* < 0.05). (d) For the SJ task, behavioral performance was plotted as the proportion of trials perceived as synchronous for test sessions completed following an infusion of aCSF (black circles) or muscimol (blue squares). Following an infusion of muscimol, a greater proportion of SJ trials was perceived as synchronous at SOAs of 40 and 200 ms (^∗∗^*p* < 0.01) and a trend towards an increase was observed at an SOA of 100 ms (*p* = 0.08). Results are displayed as mean ± SEM for the rats trained to perform the TOJ (*n* = 8) and SJ (*n* = 8) tasks.

Prior to commencing behavioral training, rats were weighed daily and maintained on a food-restricted diet until they neared 85% of their free-feeding body mass. Over the course of several stages of training, rats learned to associate a given audiovisual stimulus condition with a specific feeder (i.e., TOJ task: auditory-first = left trough and visual-first = right trough; SJ task: synchronous = left trough and asynchronous = right trough; Figure 1(a)). Once rats successfully reached the final stage of training, they were able to accurately discriminate between auditory and visual stimuli presented at an SOA of ±200 ms for the TOJ task and synchronous (i.e., 0 ms SOA) versus asynchronous audiovisual stimuli (i.e., 200 ms SOA) in the SJ task. Throughout all stages of the behavioral training procedure, correct feeder trough responses were reinforced with a sucrose pellet and incorrect responses resulted in the house light turning off for up to 15 s, during which time a new trial could not be initiated. A full description of the behavioral training procedure can be found in our earlier publication [25].

#### 2.1.3. Surgery and Cannulation

Once rats had successfully completed all stages of behavioral training, they were prepared for chronic implantation of bilateral guide cannulae into the V2L cortex, as this would ultimately allow for the local microinfusion of artificial cerebrospinal fluid (aCSF) or muscimol prior to behavioral test sessions. In preparation for surgery, the rats were anaesthetized with isoflurane (induction: 4%; maintenance: 2%) and body temperature was maintained at 37°C using a homeothermic heating pad (507220F; Harvard Apparatus). A subcutaneous injection of meloxicam (1 mg/kg) was administered before surgery and as needed postsurgery for pain management. Once a surgical plane of anesthesia was achieved, rats were placed in a stereotaxic frame with blunt ear bars, a midline incision was made in the scalp, and the dorsal aspect of the skull was cleaned with a scalpel blade. In an effort to improve postsurgical recovery, we elected to have the guide cannulae enter into the cortex on a dorsal-medial-to-ventral-lateral approach, as this left the temporalis muscles intact. After small burr holes were drilled into the skull, stainless steel guide cannulae (26 gauge, 3 mm in length) were bilaterally implanted to target the V2L cortex using the following coordinates: 6 mm caudal to bregma, 5.6 mm lateral to the midline, and a 10° angle (Figure 1(b)). These guide cannulae were secured to the skull using dental cement and bone screws as anchors. Stylets were placed into the guide cannulae to prevent their blockage. Finally, the skin surrounding the surgical implant was sutured and rats were allowed to recover for one week prior to undergoing experimental test sessions that included microinfusions.

#### 2.1.4. Microinfusions and Behavioral Testing of the TOJ and SJ Tasks

The rats returned to daily behavioral training after they had fully recovered from surgery, and once their performance again achieved >80% accuracy, experimental test sessions were introduced in which novel SOAs were presented (described below). Ultimately, each rat performed two experimental test sessions following the local microinfusion of either aCSF, which served as the control condition, or muscimol, a potent agonist of GABA-A receptors, which was used to silence the neuronal activity within the V2L cortex.

Microinjections were performed in awake animals using infusion cannulae that extended 1.2 mm beyond the length of the chronically implanted guide cannulae. On a testing day, a given rat received a bilateral infusion of either aCSF (0.5 *μ*L/side) or muscimol (4 mM; 0.5 *μ*L/side) into its V2L cortex before beginning the TOJ or the SJ test session. Both sides of the brain were infused simultaneously using a microinfusion pump and Hamilton syringes paired to the infusion cannula via Teflon tubing. Infusions were made over 2 minutes (0.25 *μ*L/min), and the infusion cannulae were then left in place for an additional 2 minutes to allow adequate diffusion of the drug into the V2L cortex.

During the TOJ test sessions, 7 SOAs were randomly delivered (i.e., 0, ±40, ±100, and ±200 ms) and rats performed a minimum of 10 trials at each of the novel SOAs. For the SJ test sessions, 5 SOAs were randomly delivered (i.e., 0, 10, 40, 100, and 200 ms) and rats were presented with at least 18 trials at each of the novel SOAs. For both behavioral tasks, 70% of the trials presented consisted of training stimuli (i.e., TOJ task: ±200 ms SOA and SJ task: 0 and 200 ms SOA), while the remaining 30% of the trials was made up of the random presentation of the novel SOAs. This distribution of trials has been previously shown to reduce the potential of developing a side bias [25]. Furthermore, the trained stimulus conditions continued to be positively reinforced for correct responses with sucrose pellets and punished for incorrect responses with a 15 s timeout, whereas a sucrose pellet was delivered following each novel SOA regardless of whether a correct or incorrect response was made.

For each of the TOJ test sessions, performance across all 7 SOAs was measured as the proportion of trials in which the rat perceived the stimuli as visual first (i.e., it responded to the right feeder trough; Figure 1(a)). Consistent with human testing, a psychophysical profile was generated for each rat by plotting straight lines between each of the neighboring SOAs and the associated slope and intercept values were calculated [26]. Using these values, the point of subjective simultaneity (PSS) and just noticeable difference (JND) was calculated for each of the test sessions [24, 25, 27]. For each of the SJ test sessions, performance for all 5 SOAs was measured as the proportion of trials in which the rat perceived the stimuli as synchronous (i.e., responded to the left feeder trough, Figure 1(a)). Similar to the TOJ task, a psychophysical profile was generated for each rat by plotting straight lines between each of the neighboring SOAs tested and the associated slope and intercept values were tabulated [24, 25]. Using these values, two audiovisual asynchrony thresholds (50% and 70%) were extracted, as these are common values used to determine the temporal binding window (TBW) in humans [21, 28–30].

### 2.2. Experiment 2: Electrophysiological Investigation of Audiovisual Temporal Processing following Noise-Induced Hearing Loss

#### 2.2.1. Hearing Assessment

In a separate group of rats (*n* = 18) from those that performed the aforementioned behavioral testing, hearing sensitivity was assessed using the auditory brainstem response (ABR), which was carried out in a double-walled sound-attenuating chamber (MDL 6060 ENV, WhisperRoom Inc., Knoxville, TN). Consistent with the study of Schormans et al. [18], rats were anesthetized with ketamine (80 mg/kg; IP) and xylazine (5 mg/kg; IP) and subdermal electrodes (27 gauge; Rochester Electro-Medical, Lutz, FL) were positioned at the vertex, over the right mastoid and on the back. Body temperature was maintained at ~37°C using a homeothermic heating pad (507220F; Harvard Apparatus, Kent, UK). Auditory stimuli consisted of a click (0.1 ms) and two tones (4 kHz and 20 kHz; 5 ms duration and 1 ms rise/fall time) which were generated using a Tucker Davis Technologies RZ6 processing module sampled at 100 kHz (TDT, Alachua, FL). Auditory-evoked activity was collected using a low-impedance headstage (RA4LI; TDT), preamplified and digitized (RA16SD Medusa preamp; TDT), and sent to a RZ6 processing module via a fiber optic cable. Stimulus delivery and threshold detection were performed in accordance with an established protocol [18, 19]. The sound stimuli used in the ABR testing, as well as the subsequent noise exposure and electrophysiological recordings, were calibrated using custom MATLAB software (MathWorks, Natick, MA) using a 1/4-inch microphone (2530; Larson Davis) and preamplifier (2221; Larson Davis).

Prior to the *in vivo* extracellular electrophysiological recordings, rats in the control group (*n* = 8) underwent an ABR to assess their hearing sensitivity, while rats in the noise-exposed group (*n* = 10) underwent a baseline hearing assessment, followed by exposure to broadband noise (see below for details). Two weeks following the noise exposure, a final hearing assessment was performed, after which the same electrophysiological recordings were completed as those in control rats. Electrophysiological recordings were completed two weeks following the noise exposure, as previous studies have demonstrated extensive region- and layer-specific plasticity across the higher-order sensory cortices at this time postnoise exposure [19].

#### 2.2.2. Noise Exposure

Under ketamine (80 mg/kg; IP) and xylazine (5 mg/kg; IP) anesthesia, rats were bilaterally exposed to broadband noise (0.8–20 kHz) for two hours at 120 dB SPL and body temperature was maintained at ~37°C using a homeothermic heating pad. This noise exposure was selected because it has been shown to be effective at inducing changes in the auditory cortex [31] and higher-order, multisensory cortices [18]. The broadband noise was generated with TDT software (RPvdsEx) and hardware (RZ6) and delivered by a super tweeter (T90A; Fostex, Tokyo, Japan) which was placed 10 cm in front of the rat.

#### 2.2.3. Surgical Procedure

Following the final hearing assessment, each rat was maintained under ketamine/xylazine anesthesia and fixed in a stereotaxic frame with blunt ear bars. Anesthetic depth was assessed by the absence of a pedal withdrawal reflex, and supplemental doses of ketamine/xylazine were administered IM as needed. An incision was made along the midline of the skull, and the dorsal aspect of the skull was cleaned with a scalpel blade. The left temporalis muscle was reflected and removed using a blunt dissection technique in order to provide access to the temporal bone overlying the left auditory and multisensory cortices. A stereotaxic micromanipulator was used to make a mark on the skull 6 mm caudal of bregma, which represents the approximate stereotaxic coordinates of the lateral extrastriate visual (V2L) cortex [18, 32–34]. Additional marks were made on the temporal bone at 1, 2, and 3 mm ventral of the top of the skull for later drilling. A small hole was hand drilled, and a stainless steel screw was inserted in the left frontal bone to serve as an anchor for the headpost and electrical ground. In order to provide free-field sound stimulation, a headpost was fastened to the skull with dental acrylic. Once the dental cement had hardened, a craniotomy (2 × 5 mm; 5-7 mm caudal to bregma) was made in the left temporal and parietal bones to expose the multisensory cortex. Subsequently, the right ear bar was removed to allow free-field auditory stimulation of the right ear during electrophysiological recordings in the contralateral cortex. The rat was held in position throughout the duration of the experiment within the stereotaxic frame using the left ear bar and the headpost.

#### 2.2.4. Electrophysiological Recordings and Stimulation Parameters

**Figure 2:**
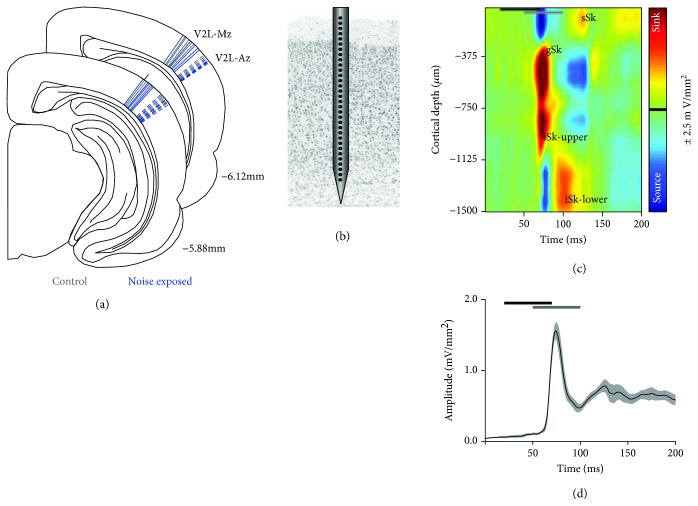
Recording site reconstruction and audiovisual-evoked CSD analysis in the multisensory zone of the V2L cortex (V2L-Mz). (a) The schematic shows a reconstruction of all recording penetrations in normal-hearing control rats (grey; *n* = 16) and noise-exposed rats (blue; *n* = 20). In each rat, two penetrations were performed in the lateral extrastriate visual cortex: one in the multisensory zone of V2L (V2L-Mz) and the other in the more ventral-positioned, auditory zone of V2L (V2L-Az). (b) A representation of a 32-channel linear electrode array spanning the entire cortical thickness within V2L-Mz. (c) Representative CSD profile in a control rat in response to audiovisual stimuli presented at an SOA of 30 ms. Prominent current sinks (red) are reflective of a depolarization of neurons in the surrounding cortical region, whereas prominent current sources (blue) reflect a repolarization of neurons in the surrounding cortical regions. The black horizontal bar denotes the presentation of the visual stimulus (50 ms LED flash at 15 lux), and the grey horizontal bar shows the timing of the auditory stimulus (50 ms noise burst at 40 dB above click threshold). (d) Average rectified current source density (AVREC) analysis derived from the CSD profiles in (c) in response to the combined audiovisual stimulus.

In each animal, two recording penetrations were performed which encompassed the majority of the audiovisual cortex. At each of the recording locations (described in detail below), a small slit was made in the dura and a 32-channel linear electrode array was slowly inserted perpendicular to the cortical surface (Figure 2(b)) using a hydraulic microdrive (FHC, Bowdoin, ME). The array consisted of 32 iridium microelectrodes equally spaced 50 *μ*m apart on a 50 *μ*m thick shank (model: A1x32-10mm-50-177-A32; NeuroNexus Technologies, Ann Arbor, MI). Initially, the electrode array was rapidly advanced into the cortex using a high-precision stereotaxic manipulator in order to penetrate the pia mater and then withdrawn to the cortical surface. Subsequently, the hydraulic microdrive was used to slowly advance the electrode array until a depth of -1500 *μ*m was reached. Slight adjustments were made based on a characteristic sharp-negative peak of the local field potential to auditory or visual stimuli (typically -350 to -450 *μ*m in depth below the pial surface) [35]. Once the appropriate depth was reached, the electrode array was allowed to settle in place for at least 45 minutes before beginning the electrophysiological recordings. Neural signals were acquired using TDT System 3 (TDT, Alachua, FL), and the local field potential (LFP) activity was continuously acquired (digitally resampled at 1000 Hz and bandpass filtered online at 1–300 Hz).

In each rat, laminar recordings were completed in two brain regions: (1) the multisensory zone of the lateral extrastriate visual cortex (V2L-Mz; corresponding to the 2 mm ventral mark made on the skull using our measurements) and (2) the auditory zone of the lateral extrastriate visual cortex (V2L-Az; 2.5 mm ventral). Consistent with previous studies demonstrating that higher-order sensory cortices occur at the intersection of the primary sensory cortices [33], V2L-Mz is located ventral to the primary visual cortex (V1) (otherwise termed “lateral”) and its neighboring region, V2L-Az, is found dorsal to the primary auditory cortex. Figure 2(a) shows a schematic of the relative position of these zones in the V2L cortex, as well the location for each of the penetrations from all of the electrophysiological experiments.

At each of the recording locations, auditory, visual, and combined audiovisual stimuli were presented using a TDT RZ6 processing module (100 kHz sampling rate) and custom MATLAB software. Auditory stimuli consisted of 50 ms noise bursts (1-32 kHz) from a speaker (MF1, TDT) positioned 10 cm from the right pinna on a 30° angle from the midline. The intensity of the auditory stimulus was customized for each rat, such that it was presented 40 dB above the rat's click threshold (control: 68.1 ± 0.9 dB SPL; noise exposed: 80.6 ± 1.4 dB SPL) as determined by the preceding hearing assessment. Visual stimuli consisted of 50 ms light flashes (15 lux; 50 ms duration) from an LED positioned adjacent to the speaker (i.e., 10 cm from the right eye). The intensity of the visual stimulus was determined using a LED light meter (model LT45, Extech Instruments, Nashua, NH). The combined audiovisual stimuli were presented at various stimulus onset asynchronies (SOAs) in which the visual stimulus was presented 50, 40, 30, 20, 10, and 0 ms before the auditory stimulus. In total, 6 stimuli conditions were presented in a randomized order, separated by an interstimulus interval of 3–5 s and each condition was presented 50 times.

#### 2.2.5. Current Source Density (CSD) Analysis

The CSD analysis provides a spatial profile of ionic flow and a measure of the total current density that enters or leaves the extracellular matrix through the cell membrane [36, 37]. A one-dimensional CSD analysis was applied to the mean LFPs recorded simultaneously across the entire cortical thickness using the following formula:

(1) $$\begin{array}{c} \text{CSD}\approx -\frac{\Phi \left(z+n\Delta z\right)-2\Phi \left(\text{z}\right)+\Phi \left(\text{z}-n\mathrm{\Delta z}\right)}{{\left(n\Delta z\right)}^{2}}, \end{array}$$

where *Φ* is the LFP, *z* is the spatial coordinate, *∆z* is the interelectrode spacing (*∆z* = 50 *μ*m), and *n* is the differentiation grid (*n* = 4) [37–40]. The CSD equation approximates the second spatial derivative of the LFPs at each time point across electrode sites. A 3-point Hamming filter was applied in order to smooth LFPs across channels before computing CSD, as described in [35]. Consistent with previous studies [35, 37–41], current sinks were positive in amplitude and sources were negative.

The CSD analysis reveals the flow of ions into and out of the neural tissue across the cortical thickness. Current sinks represent the flow of positive ions into the neural tissue from the extracellular space, which is reflective of events such as active excitatory synaptic populations and axonal depolarization [42, 43]. Current sources represent passive return currents, which corresponds to repolarization and possibly inhibition of the neighboring tissue [36, 37, 42–44]. For each of the recording locations and each of the stimulus combinations, only CSD sinks were analyzed. Current sinks were identified as being at least 3 standard deviations above the mean voltage measured during the 50 ms before the first stimulus was presented. Within both recording locations, prominent sinks were identified in the granular (−300 *μ*m < depth≥−750 *μ*m) and infragranular upper layers (−750 < depth≥−1200 *μ*m). Additional sinks were observed in the supragranular (depth≥−350 *μ*m) and infragranular lower layers (depth<−1200 *μ*m) (see Figure 2(c) for reference).

**Figure 3:**
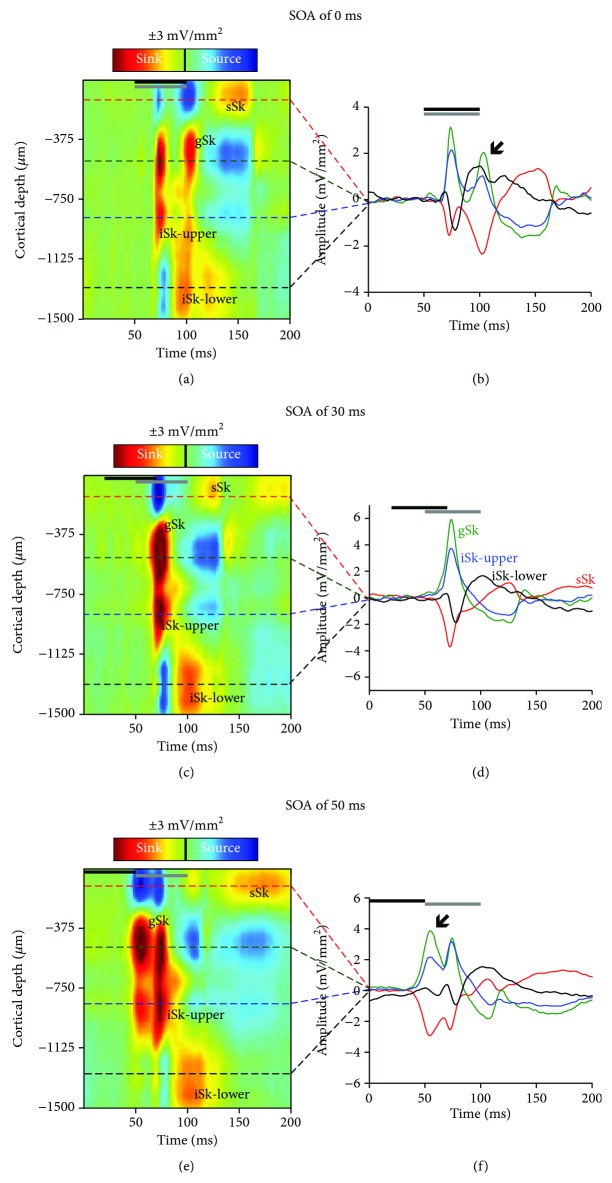
Audiovisual-evoked CSD profiles within the multisensory zone of the V2L cortex in response to 3 different SOAs. Representative CSD profiles (a, c, and e) and extracted CSD waveforms (b, d, and f) at SOAs of (a, b) 0 ms, (c, d) 30 ms, and (e, f) 50 ms in response to a combined audiovisual stimulus. CSD waveforms were extracted from the electrode showing the largest amplitude from each of the individual sinks (denoted by the dashed lines on the CSD images for the supragranular (sSk, red), granular (gSk, green), infragranular upper (iSk-upper, blue), and infragranular lower (iSk-lower, black) responses; sinks are positive, whereas sources are negative. In each of the plots, the horizontal black bar denotes the presentation of the visual stimulus (50 ms LED flash at 15 lux) and the grey horizontal bar shows the timing of the auditory stimulus (50 ms noise burst at 40 dB above click threshold). The black arrow within the CSD waveforms on panels (b,f) shows the location of the visual response, demonstrating that the visual response changes from occurring second at an SOA of 0 ms to occurring first at an SOA of 50 ms.

Consistent with Schormans et al. [19], CSD waveforms were extracted from the depth that demonstrated the largest amplitude within an individual sink (i.e., peak amplitude; see Figure 3). For each of the identified sinks, the peak amplitude was derived from a single depth in order to account for individual sink components that spanned various depths (e.g., extended beyond or were narrower than the space defined above). The peak amplitude was calculated for all stimulus combinations, and calculations were performed using custom MATLAB scripts.

#### 2.2.6. Average Rectified CSD Analysis

To examine the overall strength of postsynaptic currents in each of the cortical areas, the average rectified CSD (AVREC) measure was applied to the CSD analysis [35, 42, 45, 46]. While rectification of the CSD results in a loss of information about the direction of the transmembrane current flow, the AVREC waveform provides information about the temporal pattern of the overall strength of the postsynaptic currents [42, 47, 48]. The AVREC was calculated by averaging the absolute values of the CSD across all channels.

(2) $$\begin{array}{c} \text{AVREC}=\frac{\sum_{i=1}^{n}\left|{\text{CSD}}_{i}\right|\left(t\right)}{n}, \end{array}$$

where CSD refers to equation (1), *n* refers to the number of channels, and *t* refers to the time point index. To quantitatively analyze the AVREC waveforms for each cortical region, peak amplitude and latency were calculated for each stimulus combination within the first 200 ms from the onset of the visual stimulus (Figure 2(d)).

#### 2.2.7. Data Analysis

Multisensory interactions were quantified by comparing the response of the combined audiovisual stimulus to that of the unimodal stimulus that evoked the largest response in each experiment [49, 50]. The magnitude of the response interaction was calculated using the following formula:

(3) $$\begin{array}{c} \text{Response interaction }\left(\%\right)=\frac{\left(\text{MS}-{\text{UNI}}_{\max}\right)}{{\text{UNI}}_{\max}}\times 100, \end{array}$$

where MS is the amplitude to the combined audiovisual stimulus and UNI_max_ is the amplitude from the unimodal stimulus that evoked the largest amplitude. To analyze the temporal response profile across the various SOAs, the magnitude of the response interaction was calculated for each SOA and then averaged across experiments within each group and cortical region for both the granular sink and the AVREC amplitudes.

### 2.3. Histology

At the conclusion of both of the experimental series, the rats were injected with sodium pentobarbital (100 mg/kg; IP) in preparation for exsanguination via transcardial perfusion with 4% paraformaldehyde. Brains were serially sectioned (50 *μ*m) using a microtome (HM 430/34; Thermo Scientific, Waltham, MA). To verify that the cannulae tips were correctly located within the V2L cortex from the behavioral experiments, the coronal sections were mounted and stained with thionin. To reconstruct the location of each of the recording penetrations following the electrophysiological recordings, the coronal sections were mounted in fluorescent DAPI mounting medium (F6057 Fluoroshield™ with DAPI; Sigma, St. Louis, MO) and cover slipped. Ultimately, fluorescent and brightfield images were obtained using an Axio Vert A1 inverted microscope (Carl Zeiss Microscopy GmbH, Jena, Germany) and ZEN imaging software.

### 2.4. Statistics

Statistical analyses were conducted on the data using various procedures, including repeated-measures analysis of variance (ANOVA), one-way ANOVA, or paired/unpaired *t*-tests depending on the comparison of interest. All statistical comparisons used an alpha value of 0.05, and Bonferroni post hoc corrections were performed when appropriate. GraphPad Prism (GraphPad Software Inc.) and MATLAB (2012b; MathWorks) were used for graphical display, and SPSS (version 25, IBM Corporation) software was used for the various statistical analyses. Throughout the text and figures, data are presented as the mean values ± standard error of the mean (SEM).

## 3. Results

### 3.1. Inactivation of the V2L Cortex Shifted the Perception of Simultaneity and Perceived Synchrony

In Experiment 1, we investigated the contribution of the V2L cortex to (1) the perception of simultaneity during a TOJ task and (2) synchrony perception during an SJ task, by locally infusing the GABA-A receptor agonist, muscimol, prior to behavioral testing and ultimately comparing the performance results to those following the control condition (i.e., aCSF infusion). During the TOJ test sessions, the proportion of trials that were perceived as visual first was determined for all 7 SOAs, ranging from -200 ms (i.e., auditory-first) to +200 ms (i.e., visual-first). A two-way repeated-measures ANOVA revealed a significant interaction of infusion by SOA (*F*(4.5,31.3) = 2.8; *p* < 0.05). To further investigate this interaction, post hoc paired sample *t*-tests were completed between the test sessions. As shown in Figure 1(c), following the local microinfusion of muscimol into the V2L cortex, a significantly greater proportion of trials was perceived as visual-first at SOAs of 40 and 200 ms (*p* < 0.007), indicating that the V2L cortex plays a role in audiovisual temporal perception. Moreover, the point of subjective simultaneity (PSS), which is described as the timing at which participants are most unsure of the temporal order of the audiovisual stimuli, significantly increased following the inactivation of the V2L cortex (aCSF: 9.2 ± 6.1 ms vs. muscimol: 55.1 ± 12.5 ms; *p* < 0.01; paired sample *t*-test). Analysis of the just-noticeable difference (JND) data demonstrated that inactivating the audiovisual cortex did not impair the ability to accurately detect the audiovisual stimuli (aCSF: 69.7 ± 9.8 ms vs. muscimol: 82.0 ± 12.6 ms; *p* = 0.45). These data reveal that the inactivation of the V2L cortex via muscimol shifted the perception of simultaneity but did not affect temporal sensitivity during the TOJ task. Thus, the V2L cortex appears to play an important role in perceiving the relative timing of the audiovisual stimuli but does not influence the ability to detect subtle timing differences between the stimuli.

During the SJ test sessions, the proportion of trials that were perceived as synchronous (i.e., the rat responded to the right feeder trough) was determined for all 5 SOAs ranging from 0 ms (i.e., synchronous) to 200 ms (i.e., asynchronous; visual stimulus presented 200 ms before the auditory stimulus). Overall, a two-way repeated-measures ANOVA revealed the main effects of infusion and SOA (*F*(1, 7) = 11.1, *p* < 0.05, and *F*(1.5,10.3) = 98.8, *p* < 0.001, respectively) but no significant interaction of infusion by SOA (*F*(4, 28) = 85.4, *p* = 0.13). Follow-up paired sample *t*-tests demonstrated that a larger proportion of trials was perceived as synchronous at SOAs of 40 and 200 ms (*p* < 0.01; Figure 1(d)) following the inactivation of the V2L cortex. Although no additional comparisons reached statistical significance, trends were observed at SOAs of 10 (*p* = 0.09) and 100 ms (*p* = 0.08). In addition to the analyses completed on the SJ psychophysical curves, the 50% and 70% audiovisual asynchrony thresholds were examined. Consistent with the results observed on the SJ psychophysical curves, there was a significant increase in the 50% (aCSF: 67.8 ± 5.1 ms vs. 91.4 ± 8.6 ms; *p* < 0.05) and 70% (aCSF: 31.2 ± 5.6 ms vs. 55.7 ± 4.5 ms; *p* < 0.05) audiovisual asynchrony thresholds. These results reveal that inactivation of the V2L cortex impairs synchrony perception, such that physically asynchronous stimuli were more likely to be perceived as synchronous.

The collective results of Experiment 1 show for the first time that the V2L cortex is directly involved in the perceived timing of audiovisual stimuli. Moreover, the fact that these results confirm the importance of the V2L cortex in TOJ task performance was interesting given that our previous studies on hearing-impaired rats showed a preservation of audiovisual temporal perception despite extensive crossmodal reorganization in the V2L cortex in the weeks following noise-induced hearing loss. In considering this apparent paradox, we conducted Experiment 2 in which *in vivo* electrophysiological recordings were performed in noise-exposed rats to determine how their V2L cortex alters its responsiveness to audiovisual stimuli at varying SOAs, so as to ultimately preserve audiovisual temporal perception following hearing impairment.

### 3.2. Noise-Induced Hearing Loss

Consistent with [18, 19], crossmodal plasticity was induced by exposing rats to broadband noise at 120 dB SPL for two hours. To ensure that rats had a partial hearing loss, ABR thresholds were compared at baseline versus 2 weeks postnoise in the noise-exposed rats (*n* = 10). A two-way repeated-measures ANOVA revealed a significant difference in ABR thresholds 2 weeks postnoise exposure (*F*(1, 9) = 30.3, *p* < 0.001). Bonferroni post hoc correction testing (adjusted *p* value = 0.017) revealed a significant increase in the ABR threshold of the click (prenoise: 27 ± 1.1 dB SPL vs. postnoise: 39.5 ± 1.4 dB SPL; *p* < 0.001), 4 kHz stimulus (prenoise: 24 ± 1.5 dB SPL vs. postnoise: 44.5 ± 2.9 dB SPL; *p* < 0.001), and 20 kHz stimulus (prenoise: 12.5 ± 1.5 dB SPL vs. postnoise: 34.5 ± 6.3 dB SPL; *p* < 0.05). Prior to noise exposure, there were no differences in hearing sensitivity between the control and noise-exposed rats for any of the stimuli (one-way ANOVA; *p* > 0.05). In addition to examining ABR thresholds, the amplitude of the first wave of the ABR was used to assess the level of damage to the auditory nerve afferents caused by the noise exposure [51]. As expected, two weeks following the noise exposure, there was a significant reduction (56.5 ± 5.7%) in wave 1 amplitude (prenoise: 1.7 ± 0.08 uV vs. postnoise: 0.7 ± 0.09 uV; *p* < 0.001).

For all electrophysiological experiments, the intensity of the auditory stimulus (50 ms noise burst, 1-32 kHz) was adjusted for each rat in order to control for potential differences in hearing sensitivity among rats. To account for each rat's noise-induced hearing loss, the auditory stimulus was presented 40 dB SPL above its click threshold. As such, the auditory stimulus that was presented during the electrophysiological experiments to the noise-exposed rats was greater in comparison to the controls (noise exposed: 80.0 ± 1.4 dB SPL vs. control: 68.1 ± 0.9 dB SPL; *p* < 0.001, independent samples *t*-test).

### 3.3. Crossmodal Plasticity Increases Audiovisual Responsiveness within the Multisensory Zone of the V2L Cortex across a Range of SOAs

**Figure 4:**
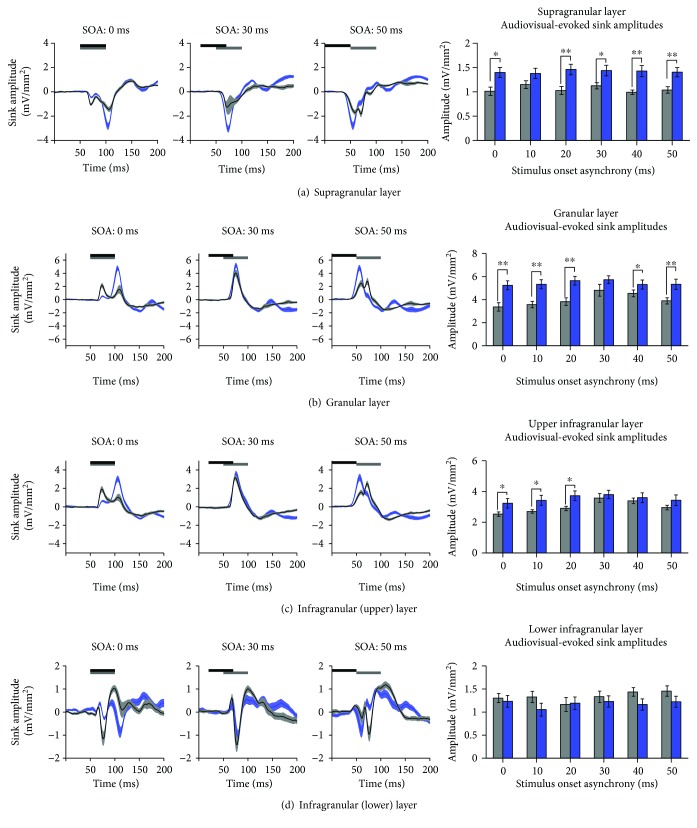
A loss of the characteristic audiovisual temporal profile was observed across the majority of layers of the multisensory zone of the V2L cortex in noise-exposed rats. Averaged CSD waveforms from the (a) supragranular, (b) granular, (c) infragranular upper, and (d) infragranular lower layers within the V2L-Mz in response to audiovisual stimuli presented at SOAs of 0, 30, and 50 ms. The black horizontal bar denotes the presentation of the visual stimulus, and the grey horizontal bar shows the timing of the auditory stimulus. The dark lines represent the group mean, and the shading represents the SEM for the noise-exposed rats (blue; *n* = 10) and age-matched controls (light grey; *n* = 8). An analysis of audiovisual-evoked sink amplitudes within each cortical layer (see bar graphs on the far right) shows an increase in responsiveness across most of the cortical layers in the noise-exposed rats. Values are displayed as mean ± SEM. ^∗^*p* < 0.05 and ^∗∗^*p* < 0.008.

Using the analysis of CSD sink amplitudes, we investigated whether noise-induced crossmodal plasticity within the multisensory zone of the lateral extrastriate visual cortex (V2L-Mz) altered audiovisual temporal processing across the cortical layers. Within V2L-Mz—a region previously shown to exhibit increased visual responsiveness following exposure to loud noise [19]—the averaged CSD waveforms were computed for both groups. Waveforms were generated for each individual sink (i.e., supragranular, granular, infragranular upper, and infragranular lower layers) in response to audiovisual stimuli presented at 6 SOAs (i.e., the visual stimulus preceded the auditory stimulus by 0, 10, 20, 30, 40, and 50 ms). Due to the large number of factors in the present study, a three-way repeated-measures ANOVA (layer × SOA × group) was performed on audiovisual-evoked CSD amplitudes within the multisensory zone of V2L (Figure 4), which ultimately revealed a significant interaction (*F*(7.9,127.0) = 3.1; *p* < 0.01). Due to the unique characteristics of each cortical sink, subsequent statistical analyses were completed for individual CSD sinks. Therefore, a separate two-way repeated-measure ANOVA (SOA × group) was performed with Bonferroni-corrected post hoc tests (adjusted *p* value = 0.008) for each of the CSD sinks.

As shown in Figure 4, there was an overall increase in audiovisual-evoked sink amplitudes across multiple SOAs and cortical layers two weeks following noise exposure. Separate two-way repeated-measures ANOVAs revealed a significant interaction of SOA by group in the supragranular layer (*F*(5, 80) = 2.6; *p* < 0.05) as well as the granular layer (*F*(5, 80) = 3.6; *p* < 0.01). Although both of the infragranular layers did not show a significant interaction, the upper infragranular layer revealed a main effect of SOA (*F*(5, 80) = 7.3, *p* < 0.001). Within the supragranular and granular layers, noise-induced hearing loss increased the level of postsynaptic activity in response to audiovisual stimulation across a range of SOAs (Figures 4(a) and 4(b)). Within the upper infragranular layer, there was a modest increase in audiovisual-evoked sink amplitudes only at SOAs less than 20 ms. Taken together, these results demonstrate that crossmodal plasticity alters audiovisual temporal processing within the multisensory zone of V2L, such that this cortical region demonstrates increased responsiveness to audiovisual stimuli across a range of SOAs.

**Figure 5:**
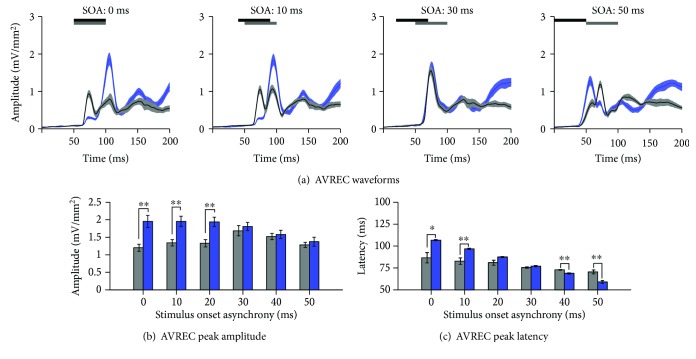
Noise-induced hearing loss enhanced the audiovisual-evoked AVREC amplitudes at select SOAs within the multisensory zone of the V2L cortex. (a) AVREC waveforms in response to audiovisual stimuli presented at SOAs of 0, 10, 30, and 50 ms (from left to right) for noise-exposed rats (blue; *n* = 10) and age-matched controls (light grey; *n* = 8). The horizontal black and grey bars denote the presentation of the visual and auditory stimuli, respectively. (b) Audiovisual-evoked AVREC amplitudes were significantly increased in the noise-exposed rats when the timing between the stimuli was less than 30 ms. (c) AVREC peak latency showed differential effects between the groups, which were dependent on the SOA. In comparison to the controls, the noise-exposed rats showed a significant increase in peak latency at SOAs less than 20 ms, whereas they showed a significant decrease in peak latency at SOAs greater than 30 ms. Values are displayed as mean ± SEM for the noise-exposed (*n* = 10) and control (*n* = 8) groups. ^∗^*p* < 0.05 and ^∗∗^*p* < 0.008.

In addition to examining the effects of noise-induced hearing loss within distinct cortical layers, AVREC waveforms were computed in order to provide additional information about the temporal pattern of the overall strength of the postsynaptic currents [42, 47, 48]. AVREC peak amplitude and latency were computed for each group in response to each of the presented SOAs. A two-way repeated-measures ANOVA revealed a significant interaction of SOA by group (*F*(5, 80) = 9.3, *p* < 0.001) for AVREC peak amplitude (Figure 5). Similar to the results observed in the upper infragranular layer, there was a significant increase in the AVREC peak amplitude at SOAs less than 30 ms (*p* < 0.008). As can be seen in Figure 5(b), SOAs from 30 to 50 ms showed no difference in peak amplitude. In order to further examine the effect of noise-induced crossmodal plasticity, AVREC peak latency was analyzed within the multisensory zone of the V2L cortex. A two-way repeated-measures ANOVA revealed a significant interaction of SOA by group for AVREC peak latency (*F*(1.6,25.7) = 19.25, *p* < 0.001). Although there was only a difference in peak amplitude at SOAs less than 30 ms, significant differences in peak latency were observed across multiple SOAs (Figure 5(c)). More specifically, there was a significant increase in latency at an SOA of 10 ms (*p* < 0.008) as well as a modest increase at an SOA of 0 ms (*p* < 0.05). Furthermore, a significant decrease in peak latency was observed at SOAs greater than 30 ms (i.e., 40 and 50 ms SOAs; *p* < 0.008). This differential response profile, whereby AVREC peak latency increases or decreases on either side of the 30 ms SOA, is consistent with the profile observed in the primary visual cortex (unpublished results from our lab). Overall, the collective results from the multisensory zone of the V2L cortex demonstrated that noise-induced crossmodal plasticity resulted in significant changes in audiovisual temporal processing across the layers of this cortical region and ultimately altered the relative timing of sensory responses after adult-onset hearing loss.

### 3.4. Audiovisual Responsiveness within the Auditory Zone of the V2L Cortex following Adult-Onset Hearing Loss

**Figure 6:**
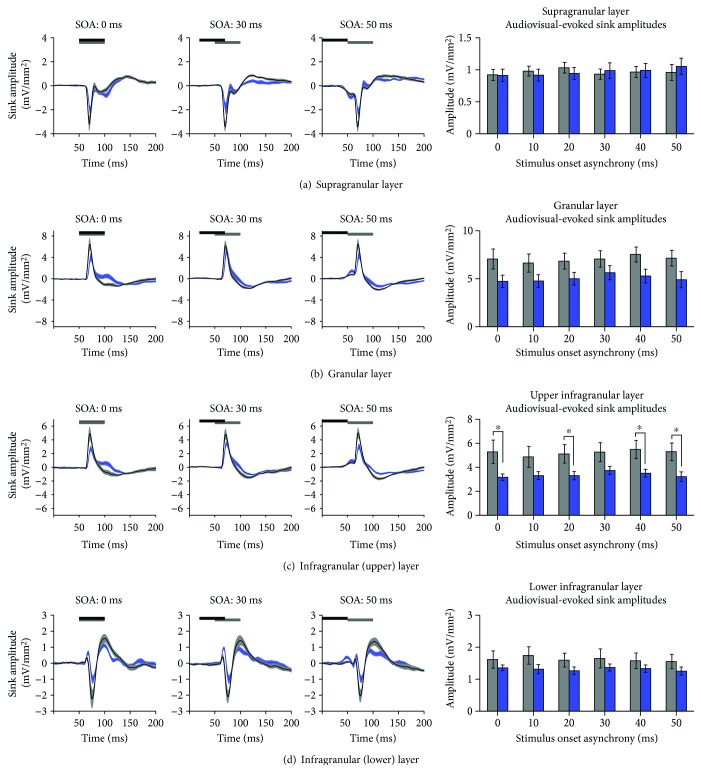
A decrease in audiovisual-evoked CSD amplitudes was generally observed within the auditory zone of the V2L cortex in noise-exposed rats. Averaged CSD waveforms from the (a) supragranular, (b) granular, (c) infragranular upper, and (d) infragranular lower layers within the auditory zone of the V2L cortex in response to audiovisual stimuli presented at SOAs of 0, 30, and 50 ms. The black horizontal bar denotes the presentation of the visual stimulus, and the grey horizontal bar shows the timing of the auditory stimulus. The dark lines represent the group mean, and the shading represents the SEM for the noise-exposed rats (blue; *n* = 10) and the age-matched controls (light grey; *n* = 8). Unlike the V2L-Mz cortex, which showed an extensive increase in the audiovisual-evoked sink amplitudes across the majority of its layers following noise-induced hearing loss (Figure 4), the auditory zone of V2L (V2L-Az) showed only a modest decrease in audiovisual responsiveness which was mostly restricted to the upper-infragranular layer (^∗^*p* < 0.05). Values are displayed as mean ± SEM.

Similar to V2L-Mz, it has been previously demonstrated that there is increased visual responsiveness within the auditory zone of the V2L cortex following noise-induced hearing loss [19]. Therefore, using the same techniques as described above, we sought to investigate whether crossmodal plasticity influenced audiovisual temporal processing across the cortical layers within a once predominantly auditory-responsive region. For each cortical layer, average CSD waveforms were computed in the two groups (control vs. noise exposure) in response to the audiovisual stimuli at multiple SOAs. A three-way repeated-measures ANOVA of audiovisual-evoked CSD sink amplitudes revealed a main effect of the cortical layer (*F*(1.6,25.2) = 72.8, *p* < 0.001). Due to the unique profile of each individual sink, subsequent statistical analyses were performed independently for each sink. Ultimately, for each of the panels in Figure 6, a separate two-way repeated-measures ANOVA (SOA × group) was performed with Bonferroni-corrected post hoc tests (adjusted *p* value = 0.008) for each of the CSD sinks.

While the multisensory zone of V2L demonstrated an overall increase in CSD sink amplitude, an opposite pattern emerged in the more ventrally located auditory zone of the V2L cortex (V2L-Az). As shown in Figure 6, there was a general decrease in the level of postsynaptic activity in response to audiovisual stimulation across a range of SOAs. Separate two-way repeated-measures ANOVAs revealed minimal differences across all of the cortical layers, as only the upper infragranular layer demonstrated a main effect of the group (*F*(1, 16) = 6.1, *p* < 0.05). Follow-up Bonferroni post hoc *t*-tests showed a modest decrease in audiovisual-evoked amplitudes across a range of SOAs (*p* < 0.05; Figure 6(c)). Overall, these results demonstrate that the multisensory zone of V2L shows the largest crossmodal effects following noise-induced hearing loss, whereas the V2L-Az cortex showed modest changes in the opposite direction.

**Figure 7:**
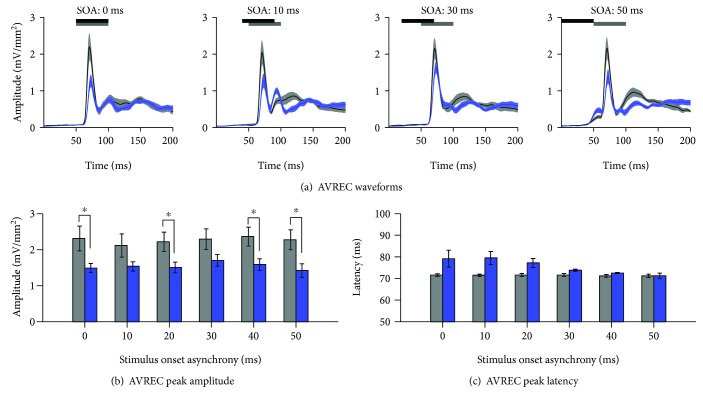
Audiovisual-evoked AVREC amplitude and latency within the auditory zone of the V2L cortex following noise-induced hearing loss. (a) Audiovisual-evoked AVREC waveforms within the auditory zone of the V2L cortex at SOAs of 0, 10, 30, and 50 ms for noise-exposed rats (blue; *n* = 10) and age-matched controls (light grey; *n* = 8). The horizontal black and grey bars denote the presentation of the visual and auditory stimuli, respectively. (b) An overall decrease in AVREC peak amplitude was observed across multiple SOAs within the auditory zone of V2L (^∗^*p* < 0.05). (c) No differences in AVREC peak latency were observed. Values are displayed as mean ± SEM.

To further examine the consequences of a partial hearing loss on the auditory zone of the V2L cortex, the overall strength of the postsynaptic currents was examined by computing AVREC waveforms for each of the groups. To do so, AVREC peak amplitude and latency were extracted from the waveforms in response to audiovisual stimuli across a range of SOAs. Overall, a two-way repeated-measures ANOVA revealed a main effect of the group (*F*(1, 16) = 4.9, *p* < 0.05) as well as a trend towards a main effect for SOA (*F*(5, 80) = 2.0, *p* = 0.08). Consistent with CSD sink amplitudes within V2L-Az, there was a general decrease in AVREC peak amplitude across multiple SOAs (*p* < 0.05; Figure 7(b)). Contrary to the multisensory zone of V2L (Figure 5(c)), the auditory zone showed no differences in peak latency (*p* > 0.05; Figure 7(c)). Therefore, despite the increased visual responsiveness observed within V2L-Az two weeks after noise-induced hearing loss, the audiovisual temporal response profile within this region was relatively maintained.

### 3.5. A Shift in the Temporal Profile following Noise-Induced Crossmodal Plasticity

To further examine changes in audiovisual processing following noise-induced hearing loss, the magnitude of response interaction was calculated for the granular sink and AVREC peak amplitudes by comparing audiovisual-evoked amplitudes to the unimodal stimulus that produced the largest amplitude. More specifically, the magnitude of response interaction for both the granular sink data and AVREC data was calculated for each group at all temporal offsets ranging from 0 ms (synchronous) to 50 ms (visual leading) within both V2L-Mz and V2L-Az. Consistent with the neuronal response profile observed in the superior colliculus [49, 50] and the V2L cortex [25] in normal-hearing animals, we expected that peak amplitudes within the multisensory zone of the V2L cortex would show the same temporal sensitivity whereby the greatest response interaction would occur when the visual stimulus preceded the auditory stimulus at SOAs of 20 to 40 ms.

**Figure 8:**
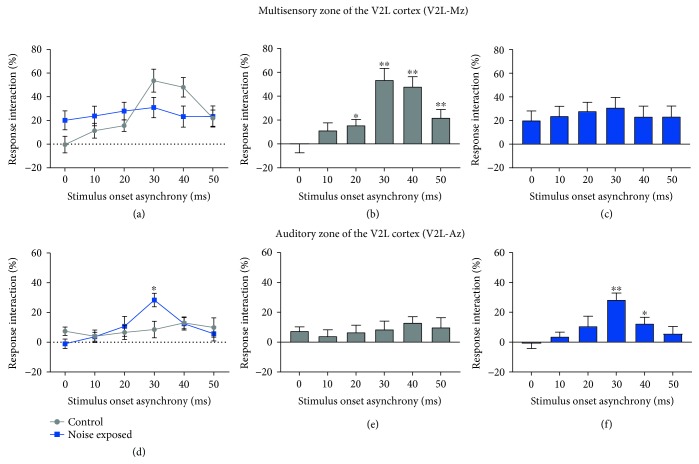
The magnitude of multisensory response interactions varied across the regions of the V2L cortex before and after noise exposure. To assess how hearing loss affected the sensitivity of neurons in the multisensory and auditory zones of the V2L cortex to the relative timing of the auditory and visual stimuli, the magnitude of the multisensory response interaction was calculated by comparing the amplitude of the granular sink in response to the combined audiovisual stimulus to that of the separately presented unimodal stimulus that evoked the largest response. Overall, a differential effect was observed between the noise-exposed rats (*n* = 10) and control rats (*n* = 8) within (a) V2L-Mz and (d) V2L-Az, with a significant difference between groups at 30 ms SOA (^∗^*p* < 0.05). (b, c, e, and f) Bar graphs show the change in the multisensory response interaction at each SOA within each group. In controls rats, only the neurons in V2L-Mz showed multisensory interactions that were sensitive to the relative timing of the auditory and visual stimuli (compare (b) and (e)). In contrast, only the neurons in V2L-Az showed a newfound temporal sensitivity after the noise-induced hearing loss (compare (c) and (f)). Following two-way repeated-measures ANOVAs, paired sample *t*-tests were completed between each SOA and 0 ms (synchrony) to investigate the temporal profile within each cortical region (^∗^*p* < 0.05 and ^∗∗^*p* < 0.01). Values are displayed as mean ± SEM.

For the granular sink dataset, an initial three-way repeated-measures ANOVA found a significant interaction of the area by SOA by group (*F*(5, 80) = 8.44, *p* < 0.001), and thus, we further examined each of these interactions in order to reveal the specific differences between the groups, as well as the temporal profiles within each of the groups. As shown in Figure 8, the response interactions in the granular layer of the multisensory zone as well as the auditory zone of the V2L cortex showed drastic differences between the noise-exposed rats and the controls. Within V2L-Mz, a significant interaction of SOA by group was observed (*F*(5, 80) = 7.02, *p* < 0.001), yet post hoc *t*-tests failed to show significant differences between the groups at any of the SOAs presented (Figure 8(a)). In contrast, within V2L-Az, a two-way repeated-measures ANOVA revealed a significant interaction of SOA by group (*F*(5, 80) = 3.82, *p* < 0.01) and post hoc *t*-tests found a difference between groups at 30 ms SOA (*p* = 0.013) in which the noise-exposed rats demonstrated an increased response interaction compared to the controls (Figure 8(d)). Next, to examine how the timing of the audiovisual stimuli influenced the response interaction in the granular layer of both groups, separate one-way repeated-measures ANOVAs were performed in the noise-exposed and control rats. As expected, the multisensory zone of V2L of control rats demonstrated a main effect of SOA (*F*(5, 35) = 13.91, *p* < 0.001) and these rats showed a significant increase in the magnitude of the response interaction at SOAs of 30, 40, and 50 ms when compared to an SOA of 0 ms (paired sample *t*-test; *p* < 0.01; Figure 8(b)). In contrast, there was no main effect of SOA in the multisensory zone of the V2L cortex of noise-exposed rats (*F*(5, 45) = 0.70, *p* = 0.624). Furthermore, the opposite pattern emerged in the auditory zone of V2L, where there was no effect of stimulus timing in controls (one-way rmANOVA (*F*(5, 35) = 0.90, *p* = 0.493)), but in the noise-exposed rats, there was a significant increase in the magnitude of the response interaction at an SOA of 30 ms (*p* < 0.01) and a modest increase at an SOA of 40 ms (*p* = 0.011). These findings highlight that the typical temporal profile observed in the granular layer of the multisensory zone of the V2L cortex in normal-hearing rats was now evident in the more ventrally located auditory zone in the noise-exposed rats.

**Figure 9:**
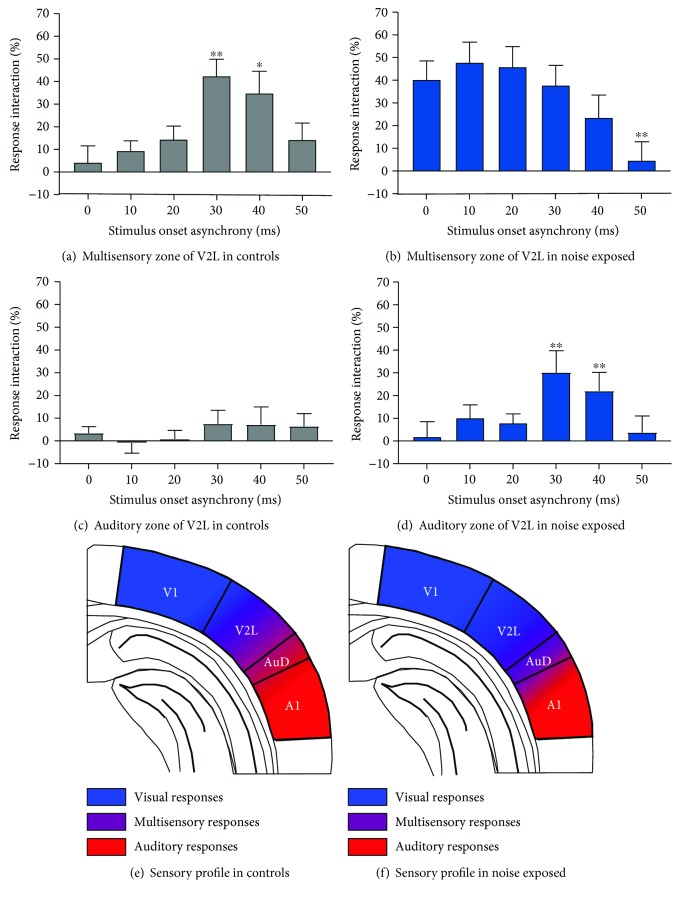
Compensatory plasticity in the auditory zone of the V2L cortex preserves audiovisual temporal processing following moderate hearing loss. Using the AVREC amplitude as a measure of the overall strength of postsynaptic currents in a given cortical region, the magnitude of the multisensory response interactions was then calculated at each SOA to determine how noise-induced hearing loss affected the sensitivity of neurons in the multisensory and auditory zones of the V2L cortex to the relative timing of the auditory and visual stimuli. Ultimately, the temporal profile observed in V2L-Mz of control rats (a), in which there was a significant increase in the magnitude of the multisensory response interaction at SOAs of 30 and 40 ms, was consistent with the temporal profile that emerged within V2L-Az of noise-exposed rats (d). Following two-way repeated-measures ANOVAs, paired sample *t*-tests were completed between each SOA and 0 ms (synchrony) to investigate the temporal profile within each cortical region (^∗^*p* < 0.05 and ^∗∗^*p* < 0.01). Values are displayed as mean ± SEM. (e, f) As schematized, it appears that noise exposure did not result in a loss of temporally precise audiovisual processing but instead caused a functional transition in the cortical region displaying this temporal sensitivity—findings which are suggestive of compensatory plasticity having occurred following moderate hearing loss.

Additional support for a functional transition in the cortical region showing the greatest degree of audiovisual response interaction was evident from analyses of the AVREC data collected from the multisensory and auditory zones of the V2L cortex in noise-exposed rats versus controls. As shown in Figure 9, the influence of the SOA on the degree of response interaction in the multisensory zone of the V2L cortex was evident in the control rats (Figure 9(a) (*F*(5, 35) = 8.51, *p* < 0.001)) but not in the noise-exposed rats (Figure 9(b)), as they failed to show a preferred response interaction when the visual stimulus preceded the auditory stimulus by 30 ms compared to when they were presented simultaneously (0 ms SOA). That said, the auditory zone of the V2L cortex of the noise-exposed rats, unlike the controls, now showed evidence of temporal sensitivity in the magnitude of the response interaction (Figure 9(d) (*F*(5, 45) = 7.72, *p* < 0.001). Interestingly, when paired sample *t*-tests were completed between each SOA and 0 ms (synchrony), a consistent profile emerged between V2L-Mz in the controls (Figure 9(a)) and V2L-Az in the noise-exposed rats (Figure 9(d)), in which both regions showed a significant increase in the magnitude of the response interaction of the AVREC at SOAs of 30 ms (*p* < 0.05) and 40 ms (*p* < 0.01). Thus, these collective results are consistent with a functional transition in the cortical region showing the greatest degree of audiovisual temporal sensitivity following adult-onset hearing loss (Figures 9(e) vs.  9(f)).

## 4. Discussion

Following moderate hearing loss, neurons in the auditory cortex as well as the higher-order audiovisual cortex maintain a residual capacity for sound processing, while also now demonstrating crossmodal plasticity, a phenomenon characterized by an increased responsiveness to visual stimuli [15, 17]. Interestingly, despite this sensory reorganization, behavioral studies on hearing-impaired humans and rats have reported that audiovisual temporal acuity—the perceptual ability to accurately judge the relative timing of auditory and visual stimuli—is largely unaffected [21, 24]. To investigate the potential neurophysiological basis of how audiovisual temporal acuity may be preserved in the presence of hearing loss-induced crossmodal plasticity, we exposed adult rats to loud noise and two weeks later performed *in vivo* electrophysiological recordings across the distinct layers of neighboring regions of the audiovisual cortex (i.e., the lateral extrastriate visual area (V2L)) to ultimately assess the nature and extent of changes in audiovisual temporal processing at the level of postsynaptic potentials. In particular, we sought to determine whether the increased visual responsiveness of neurons in a once predominantly auditory area was also accompanied by a newfound capacity to temporally integrate auditory and visual information similar to that of the audiovisual cortex in normal-hearing rats—electrophysiological results that could provide the neural substrate for the preservation of audiovisual temporal perception following adult-onset hearing loss.

### 4.1. The Role of the Lateral Extrastriate Visual (V2L) Cortex in Audiovisual Temporal Processing and Perception

Previous studies on normal-hearing rats have reported that the V2L cortex, which is wedged between the primary visual cortex (V1) and the dorsal auditory cortex (AuD), shows several hallmarks of cortical multisensory processing consistent with other mammals [25, 32–34, 52, 53]. For example, within the V2L cortex, there exists a diverse population of sensory-responsive neurons, some of which show robust spiking responses to both auditory and visual stimuli (i.e., bimodal neurons) and others that only overtly respond to a single modality, yet this response can be modulated by the other seemingly ineffective modality (i.e., subthreshold multisensory neurons) [18]. Moreover, in normal-hearing rats, the cortical region that has the greatest proportion of bimodal neurons (i.e., the V2L multisensory zone (V2L-Mz)) is relatively small (~500 *μ*m span from dorsal to ventral), whereas the areas flanking V2L-Mz, such as the auditory or visual zones of the V2L cortex (V2L-Az and V2L-Vz, respectively), have a reduced capacity for multisensory processing [18, 33]. To further investigate the multisensory profile of the V2L cortex, in the present study, we simultaneously recorded the LFP activity across the distinct layers of V2L-Mz and V2L-Az in response to separate versus combined auditory and visual stimulation at various temporal offsets. As expected, the subsequent CSD analyses revealed that neurons in V2L-Mz showed the greatest multisensory response interaction when the visual stimulus preceded the auditory stimulus by ~30-40 ms (Figures 8(b) and 9(a)), whereas the neurons in V2L-Az did not show any preferential multisensory effects upon manipulation of the relative timing of the auditory and visual stimuli (Figures 8(e) and 9(c)). Thus, in normal-hearing rats, audiovisual temporal processing appeared to be restricted to a discrete region of the higher-order multisensory cortex (Figure 9(e)).

Based on these electrophysiological findings, it would be reasonable to suspect that the V2L cortex plays a role in perceptual tasks that require audiovisual temporal acuity, such as those in which the rats must judge the temporal order of auditory and visual stimuli (TOJ task) or whether the auditory and visual stimuli were presented synchronously or not (SJ task). To investigate this possibility, we chronically implanted cannulae into the V2L cortex of normal-hearing rats that had been trained to perform the TOJ or SJ task and then microinfused muscimol (or aCSF) prior to behavioral testing to determine the effect of pharmacological silencing of the V2L cortex on audiovisual temporal acuity. Ultimately, this novel experimental series revealed that the inactivation of the V2L cortex (1) caused a shift in the perception of simultaneity during the TOJ task, such that the light flash now had to be presented much earlier before the noise burst for the two stimuli to be perceived as having occurred simultaneously, and (2) caused a lengthened epoch of time over which the physically asynchronous auditory and visual stimuli were perceived to have occurred at the same moment in time (i.e., the temporal binding window increased on the right side of physical synchrony) (Figure 1). Taken together, these findings confirm that the V2L cortex contributes to audiovisual temporal acuity and ultimately prompted us to wonder what happens at the neuronal level to audiovisual temporal processing in the V2L cortex following noise-induced hearing loss that allows for audiovisual temporal perception to be preserved in the presence of crossmodal plasticity.

### 4.2. Effects of Hearing Loss on Audiovisual Temporal Processing

Our previous studies on noise-exposed rats found a significant reduction in the auditory-evoked activity in V2L-Mz (despite increasing the noise burst intensity to control for their elevated hearing thresholds) and a concomitant increase in visual responsiveness in the neighboring region, V2L-Az [18, 19]. Consequently, in the present study, we predicted that, in addition to showing increased multisensory convergence postnoise exposure, neurons in V2L-Az would also be able to process audiovisual stimuli with the temporal selectivity that was previously restricted to V2L-Mz in normal-hearing rats. In support of this prediction, we found a differential effect of hearing loss-induced crossmodal plasticity in the neighboring regions of the V2L cortex, whereby the typical temporal profile observed in the granular layer of V2L-Mz in normal-hearing rats (i.e., an increased multisensory response interaction when the visual stimulus preceded the auditory stimulus by ~30 ms; Figure 8(b)) was now only present in the more ventrally located V2L-Az in the noise-exposed rats (Figure 8(f)). Thus, we have shown for the first time that hearing loss-induced crossmodal plasticity does not result in a loss of temporally precise audiovisual processing, but instead, there appears to be a functional transition in the cortical region displaying this temporal sensitivity (schematized in Figures 9(e) and 9(f)).

At present, the cellular mechanisms underlying the functional shift in multisensory convergence across the neighboring cortical regions remain elusive. With respect to hearing loss-induced crossmodal plasticity in general, it has been postulated that cortical reorganization may emerge via (1) altered multisensory processing in subcortical loci that ultimately manifests as cortical plasticity [54], (2) a loss of local cortical inhibition [55], (3) altered dendritic spine density in the deprived cortical region [56], and/or (4) a complex assortment of homeostatic plasticity associated with the upward and downward scaling of intracortical and thalamocortical excitatory synapses in the deprived and spared cortices [57–60]. Clearly, future studies are needed to resolve which, if any, of the aforementioned mechanisms contribute to the transition in the functional boundary of the audiovisual cortex following moderate hearing loss in adulthood. We suspect, however, that this *functional* transition of the audiovisual cortex would not likely be due to an *anatomical* shift in the territorial borders of the respective cortices because no significant differences in the cytoarchitectonic borders and cortical connectivity were found within the sensory cortices of congenitally deaf cats [61–63]—a much more extreme model of sensory deprivation than the moderate hearing impairment induced in the present study.

### 4.3. Compensatory Plasticity following Hearing Loss

To date, the vast majority of studies that have investigated the behavioral consequences of hearing loss-induced crossmodal plasticity have focused on humans and laboratory animals with profound hearing loss. Given the improved processing of peripheral visual stimuli and visual motion [7–11] commonly reported in these deaf subjects, the underlying neurophysiological changes have been described as “compensatory” in nature. To our knowledge, the present study provides the first evidence of *compensatory plasticity* at the neuronal level following moderate hearing loss, characterized by a transition in the functional boundary of the audiovisual cortex that ultimately preserved the temporal sensitivity of multisensory processing postnoise exposure. Based on these neurophysiological results, it is reasonable to postulate that this compensatory plasticity ultimately contributes to the preservation of audiovisual temporal acuity previously reported in humans and rats with hearing impairment [21, 24].

## 5. Conclusions

The present study is aimed at advancing our understanding of the nature and extent of sensory reorganization that occurs following moderate hearing loss in adulthood, with an emphasis on how this highly prevalent form of sensory deprivation impacts audiovisual temporal processing at the neuronal level. Using a rat model of noise exposure and layer-specific electrophysiological recordings of postsynaptic potentials in neighboring regions within the lateral extrastriate visual (V2L) cortex, we have shown for the first time that adult-onset hearing loss does not result in a loss of temporally precise audiovisual processing but rather a shift in the cortical region displaying this capacity for temporal sensitivity. Indeed, although the neurons in the multisensory zone of the V2L cortex of noise-exposed rats no longer showed the canonical enhancement of multisensory responses when the visual stimulus preceded the auditory stimulus by ~30 ms, this temporal profile emerged in the neighboring cortical region, the once predominantly auditory zone of V2L. Future studies are needed to uncover the cellular mechanisms associated with this compensatory plasticity and whether the transition in the functional boundary of the audiovisual cortex is indeed the neural substrate for the preservation of audiovisual temporal perception reported in hearing-impaired subjects.

## Data Availability

The electrophysiological data used to support the findings of this study are available from the corresponding author upon request.

## Abbreviations

ABR: Auditory brainstem response
PSS: Point of subjective simultaneity
aCSF: Artificial cerebrospinal fluid
rmANOVA: Repeated-measures ANOVA
ANOVA: Analysis of variance
SEM: Standard error of the mean
AuD: Dorsal auditory cortex
SJ: Synchrony judgment
AVREC: Average rectified current source density
SOA: Stimulus onset asynchrony
CSD: Current source density
sSk: Supragranular sink
dB SPL: Decibels, sound pressure level
TBW: Temporal binding window
gSk: Granular sink
TOJ: Temporal order judgment
IM: Intramuscular
V1: Primary visual cortex
IP: Intraperitoneal
V2L: Lateral extrastriate visual cortex
iSk-lower: Infragranular sink-lower
V2L-Az: Auditory zone of the lateral extrastriate visual cortex
iSk-upper: Infragranular sink-upper
V2L-Mz: Multisensory zone of the lateral extrastriate visual cortex
JND: Just noticeable difference
LFP: Local field potential.
